# Interferon antagonists encoded by SARS-CoV-2 at a glance

**DOI:** 10.1007/s00430-022-00734-9

**Published:** 2022-04-02

**Authors:** Jung-Hyun Lee, Lennart Koepke, Frank Kirchhoff, Konstantin M. J. Sparrer

**Affiliations:** 1 Institute of Molecular Virology, Ulm University Medical Center, Meyerhofstr. 1, 89081 Ulm, Germany; 2 Present Address: Department of Life Science, University of Seoul, Seoul, 02504 Republic of Korea

**Keywords:** Interferon, SARS-CoV-2, Innate immunity, COVID-19, Immune evasion

## Abstract

The innate immune system is a powerful barrier against invading pathogens. Interferons (IFNs) are a major part of the cytokine-mediated anti-viral innate immune response. After recognition of a pathogen by immune sensors, signaling cascades are activated that culminate in the release of IFNs. These activate cells in an autocrine or paracrine fashion eventually setting cells in an anti-viral state via upregulation of hundreds of interferon-stimulated genes (ISGs). To evade the anti-viral effect of the IFN system, successful viruses like the pandemic severe acute respiratory syndrome coronavirus 2 (SARS-CoV-2) evolved strategies to counteract both IFN induction and signaling. In fact, more than half of the about 30 proteins encoded by SARS-CoV-2 target the IFN system at multiple levels to escape IFN-mediated restriction. Here, we review recent insights into the molecular mechanisms used by SARS-CoV-2 proteins to suppress IFN production and the establishment of an anti-viral state.

## Introduction

Invading viruses are detected by pattern recognition receptors (PRRs) like RIG-I-like receptors (RLRs) and Toll-like receptors (TLRs) recognizing viral pathogen-associated molecular patterns (PAMPs) [1]. For example, infection with severe acute respiratory syndrome coronavirus 2 (SARS-CoV-2), the pathogen that causes the current coronavirus disease 2019 (COVID-19) pandemic, is recognized by various PRRs, most prominently the RLR melanoma differentiation-associated protein 5 (MDA5) and TLRs, such as TLR2 [2], 3 [3] and 4 [4]. The exact nature of the SARS-CoV-2 PAMP(s) is currently unknown. However, it was suggested that the Spike protein may mediate TLR2 and TLR4 activation [2, 5, 6]. After recognition of a pathogen by PRRs, downstream signaling cascades are induced that ultimately lead to the activation of a kinase called Tank-binding kinase 1 (TBK1) that mediates phosphorylation of a set of transcription factors called interferon regulatory factors (IRFs), among them IRF3 and IRF7. Dimerization and translocation of IRFs to the nucleus eventually induce the expression and subsequent release of various (pro-)inflammatory cytokines, most prominently interferons (IFNs). There are three major types of IFNs, type I, type II, and type III, classified by their receptor usage. While type I and III IFNs are released by most nucleated cell types upon PRR activation, type II IFNs are mainly produced by activated T cells and natural killer cells [7]. Type I IFNs include primarily IFN-α (comprising 13 different subtypes) and IFN-β, but also more recently IFN-ε/κ, IFN-ω, and IFN-ν [8]. The only cytokine classified as type II IFN is IFN-γ. Three type III IFNs, IFN-λ1, IFN-λ2, and IFN-λ3, were formerly known as Interleukins (IL) IL29, IL28A, and IL28B with overlapping but distinct functions compared to type I IFNs [9]. Notably, receptors for type III IFNs are more specifically expressed on epithelial cells and some types of immune cells, such as dendritic cells and neutrophils, whereas type I and II IFN-receptors are present on almost all nucleated cells [10, 11]. Binding of IFNs in either a paracrine or autocrine fashion to their respective receptors results in the activation of signal transduction kinases, among them janus kinases (JAK) and tyrosine kinases (TYK) [12, 13]. These kinases in turn activate members of the signal transducer and activator of transcription (STAT) protein family, that drive the expression of hundreds of interferon-stimulated genes (ISGs) [14], many of which are known to restrict viral replication and the spread of viruses [12, 13, 15]. Thus, an anti-viral state in both virus-infected cells and uninfected bystander cells [8, 13, 16] is induced. In addition to activating the innate immune defenses, IFNs also play an important role in recruiting and activating cells of the adaptive immune system. However, successful viruses like SARS-CoV-2 have evolved strategies to evade or counteract the induction, signaling, and anti-viral effects of IFNs [17–19]. In this review, we provide a brief overview of how SARS-CoV-2 uses many of its proteins to counteract IFN induction and signaling, thus preventing effective innate immune activation.

## Main text

The 30 kb positive-sense single-strand RNA genome of SARS-CoV-2 encodes approximately 30 proteins [20]. Sixteen non-structural proteins (Nsp1-16) are produced by (auto-)proteolytic processing of two large precursor polyproteins open reading frame 1a (ORF1a) and ORF1ab, which are both translated from the full-length SARS-CoV-2 RNA. ORF1ab arises due to a ribosomal frameshift, allowing the translation to continue beyond the ORF1a stop codon. From subgenomic mRNAs, SARS-CoV-2 expresses four structural proteins, namely spike (S), envelop (E), membrane (M), and nucleocapsid (N), and several accessory factors. Among the accessory proteins, are ORF3a, ORF3b, ORF6, ORF7a, ORF7b, ORF8, ORF9b, and ORF10. Notably, some genes may encode for several proteins, such as the ORF3 locus which codes for ORF3a, b and possibly other products, like ORF3c [21]. Classically, viruses use their accessory proteins to counteract innate immune activation or effectors. However, recent reports have established that SARS-CoV-2 uses its non-structural, structural, as well as accessory proteins to counteract IFN induction and signaling [17–19].

### Non-structural proteins

**Fig. 1:**
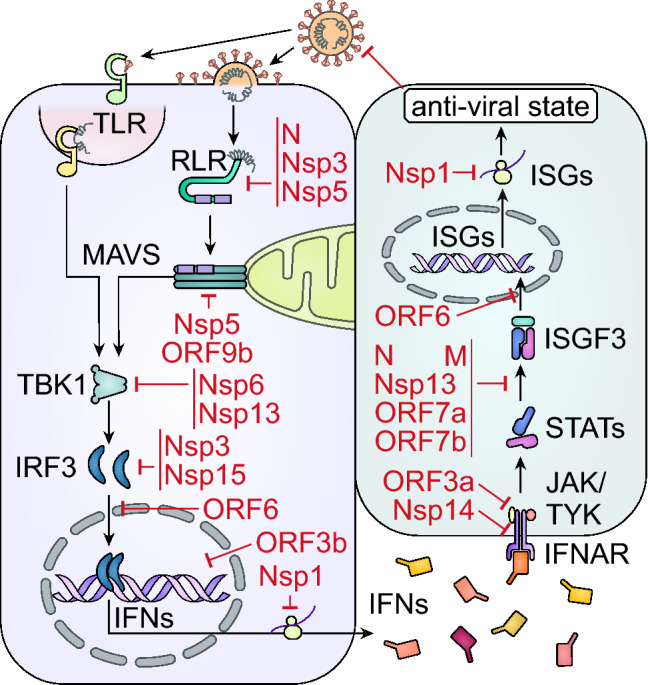
Counteraction of the IFN system by SARS-CoV-2 proteins. Schematic depiction of the antagonism of the interferon (IFN) system by severe acute respiratory syndrome coronavirus 2 (SARS-CoV-2) proteins. Incoming or replicating virus is recognized by Toll-like receptors (TLRs) or RIG-I-like receptors (RLRs) which eventually activates interferon regulatory factor 3 (IRF3) either through TANK binding kinase protein 1 (TBK1) or via mitochondrial antiviral-signaling protein (MAVS). Activated IRF3 dimerizes and translocates to the nucleus, where it induces the production of IFNs. IFNs bind to their respective receptors (e.g., interferon alpha and beta receptor subunit 1, IFNAR) to induce janus kinase (JAK) and tyrosine kinase (TYK) mediated activation of signal transducer and activator of transcription (STATs). Activated STAT complexes (ISGF3) translocate to the nucleus, where they induce transcription of interferon-stimulated genes (ISGs). Induction of ISGs sets the cell in an antiviral state that restricts infection and replication of the virus. SARS-CoV-2 interferes with signal transduction at multiple levels, as indicated by red highlights. *Nsp* non-structural protein, *N* nucleocapsid protein, *M* matrix protein, *ORF* open reading frame

The 16 non-structural proteins encoded by SARS-CoV-2 are not part of the virion but are essential for viral replication and transcription. For example, they are involved in the formation of replication complexes and play a prominent role in IFN escape, with several directly targeting key players of the IFN signaling cascade (Table 1, Fig. 1). All Nsps, except Nsp2, 4, 7–11, and 16, were reported to diminish IFN induction and/or signaling [17, 22, 23]. Notably, Nsps often have enzymatic function, such as the two main proteases of SARS-CoV-2 Nsp3 and Nsp5.

**Table 1:** Overview of SARS-CoV-2 encoded proteins and their impact on the IFN system

Protein name	Molecular mechanism	References
Nsp1	Prevents cellular translation, including ISG and cytokine expression by blocking the ribosome	[24, 25]
Nsp3	Removes activating ISGylations from MDA5 and IRF3	[29, 30]
Nsp5	Processes RIG-I and promotes proteasome-mediated degradation of MAVS	[31, 32]
Nsp6	Interacts with TBK1 to inhibit the activation of IRF3	[18]
Nsp12	Conflicting data, probably not an inhibitor	[17, 19, 33, 34]
Nsp13	Inhibits of STAT1, STAT2 and TBK1 phosphorylation/activation	[18, 19, 35]
Nsp14	Caps viral mRNAs and degrades IFNAR1	[17, 19, 35]
Nsp15	Removes PAMPs and interferes with the activation of IRF3	[38, 22]
N	Targets RLRs and prevents STAT phosphorylation	[41]
M	Decreases activation of STAT1	[19]
ORF3a	Interferes with JAK activation by elevating SOCS1 levels	[43]
ORF3b	Inhibits IFN induction/signaling via an unknown mechanism	[44]
ORF6	Reduces nuclear translocation of IRF3 and STATs via dislocating Nup98	[19, 46–48]
ORF7a and ORF7b	Prevent efficient STAT2 phosphorylation, activity is promoted by ubiquitination of ORF7a	[18, 49]
ORF9b	Interferes with RLR signaling by targeting MAVS	[50]

Nsp1 is a 180-amino acid protein, consisting of a globular domain and a C-terminal helix–turn–helix motif. It binds to the cellular ribosome plugging the mRNA entry channel with its helix–turn–helix motif thereby preventing translation of mRNAs [24, 25]. Consequently, Nsp1 drastically reduces the expression of all types of IFNs and ISGs [24]. Notably, SARS-CoV-2 replicon systems lacking functional Nsp1 are more susceptible toward type I IFNs [26].

With a size of more than 200 kDa, Nsp3 is the largest protein among the Nsps. It is a key component of the viral replication and transcription complex that assembles on host-cell membranes. Nsp3 has papain-like protease (PLpro) activity, which is required for Nsp1-4 processing, and in addition functions as a deubiquitinase and deISGylase [27, 28]. Recent studies have shown that Nsp3 antagonizes ISGylation of MDA5 thereby blocking the activation of this PRR [17, 29]. In addition, Nsp3 downregulates signal transduction by deISGylating the transcription factor IRF3 [30].

The main protease of SARS-CoV-2, Nsp5 was reported to suppress induction of type I IFN expression and signaling induced by all types of IFNs [17, 31]. Mechanistically, it has been suggested that Nsp5 cleaves retinoic acid-inducible gene I (RIG-I) rendering it inactive and promotes proteasome-mediated destruction of the signaling adaptor mitochondrial anti-viral-signaling protein (MAVS) [32]. Thus, inhibition of Nsp5 protease activity could at least partially alleviate its impact on type I IFN induction.

Nsp6 (together with Nsp3 and 4) is involved in the formation of the SARS-CoV-2 replication compartment made up of double-membrane vesicles. In addition, it was reported that Nsp6 interacts with TBK1 to inhibit activation of IRF3, in turn restricting type I IFN induction [18].

The active subunit of the SARS-CoV-2 RNA-dependent RNA polymerase Nsp12 has been suggested to suppress type I IFN induction by inhibiting nuclear translocation of the transcription factor IRF3 [33]. However, several studies by other labs did not confirm this, as Nsp12 was not picked up in various screening approaches or did explicitly not affect endogenous type I IFN induction [17, 18, 34].

The SARS-CoV-2 encoded helicase Nsp13 inhibits type I IFN production and signaling by preventing the activation of STAT1 and STAT2, which are crucial for IFN signaling [18, 19, 35]. Furthermore, immunoprecipitation experiments combined with proteomic approaches suggest that Nsp13 may bind to TBK1 to inhibit its phosphorylation and thus activation of IRF3 [18, 22, 36].

Nsp14 is a guanine-N7-methyltransferase required for efficient SARS-CoV-2 transcription. It facilitates the formation of the mRNA cap, thus preventing the recognition of SARS-CoV-2 mRNAs by PRRs [37]. In addition, Nsp14 significantly reduced IFN-β promoter-driven luciferase activities and induced degradation of endogenous interferon alpha and beta receptor subunit 1 (IFNAR1) at the protein level, thus inhibiting type I IFN binding to cells and subsequent signaling [17, 19, 35].

Nsp15 is an uridine-specific endoribonuclease [38], which was suggested to remove and/or process viral RNA that would otherwise trigger detection of the virus and thus IFN induction. Furthermore, it was reported to reduce type I IFN production by interaction with ring finger protein 41 (RNF41), an E3 ligase associated with the activation of IRF3 [22].

While many molecular mechanisms of SARS-CoV-2 Nsps have been identified, future studies are needed to evaluate the various roles of many Nsps in viral replication and IFN antagonism. Furthermore, many Nsps function in complexes, which were not analyzed in the context of IFN induction/signaling antagonism yet. Importantly, some Nsps require enzymatic function to antagonize the IFN system and may be targeted, e.g., by small molecules. For example, orally available inhibitors of Nsp5 are already examined in clinical trials [39, 40]. These inhibitors may not only restrict SARS-CoV-2 via prevention of the cleavage of its protein products but also by increasing anti-viral immune responses.

### Structural proteins

SARS-CoV-2 encodes four structural proteins on subgenomic mRNAs that are part of the assembled and infectious virion: S, N, M, and E. Recent studies reported that even the structural proteins of SARS-CoV-2 may play a role in antagonizing IFN production and signaling (Table 1). It has been shown that the N protein inhibits both type I IFN production and signaling by targeting RLRs [41] and decreasing the phosphorylation of STAT1 and STAT2 [39], respectively. In addition, the N protein wraps the genomic RNA, shielding it from recognition by PRRs. The M protein was found to suppress type I IFN induction upon Sendai virus (SeV) stimulation, or stimulation via expression of MDA5 or RIG-I [17, 19].

In addition to their structural functions as part of the virion, N and M play active roles in inhibiting the type I IFN system. Thus, future studies could investigate whether incoming virions may be sufficient to reduce type I IFN induction and signaling immediately after infection.

### Accessory proteins

Besides non-structural proteins and structural proteins coronaviruses such as SARS-CoV-2 encode for accessory proteins. Although the roles of these proteins are not fully understood, it is known that they often play crucial roles in virus–host interactions, specifically with the innate immune system (Table 1).

The ORF3 locus encodes multiple proteins, ORF3a being the longest with smaller products, like ORF3b and ORF3c, produced from downstream start codons. ORF3a was suggested to interfere with proper activation of JAKs by inducing Suppressor of Cytokine Signalling 1 (SOCS1), thus attenuating IFN signaling [43]. ORF3b was reported to inhibit IFN production through its C-terminus [44]. Interestingly, some naturally occurring SARS-CoV-2 variants encode a longer version of ORF3b with increased activity, while other strains express a truncated and presumably inactive variant of this protein [45].

Despite its small size of only 7 kDa, ORF6 was consistently identified as a very potent inhibitor of type I and/or III IFN induction and signaling [17–19, 35]. Mechanistically, it was reported that ORF6 binds to nucleoporin 98 (Nup98) dislocating it from the nuclear pore [46], thus preventing efficient nuclear import of STATs [47] and IRF3 [19, 48], in turn inhibiting both type I IFN induction and signaling.

Two proteins are expressed from the ORF7 locus: ORF7a and ORF7b. While ORF7a is a 121 aa-long transmembrane protein, ORF7b encompasses only 43 aa. Recent evidence indicates that both ORF7a and ORF7b block STAT2 phosphorylation, thereby inhibiting type I IFN signaling [18]. Curiously, the modification of ORF7a by covalently conjugated ubiquitin promotes its function as an antagonist of type I IFN responses [49].

ORF9b is a small 97 aa protein expressed from an alternative ORF in the N locus. It was suggested that it suppresses type I IFN induction by targeting the MAVS signalosome [50].

Overall, the accessory proteins clearly play a major role in antagonizing activation of the IFN system. However, it seems that antagonism of innate immunity is only a part of their function, and many of them have additional roles, e.g., in virion assembly and egress of the virus.

## Concluding remarks

IFNs are a key component of the innate immune system that set cells in an anti-viral state by inducing hundreds of (anti-viral) ISGs. Evidently, the unfortunate success of SARS-CoV-2 is critically dependent on its ability to counteract both IFN induction and signaling to evade the host’s innate defenses. Since the discovery of SARS-CoV-2 in late 2019, our knowledge about this pathogen has literally exploded and is still rapidly expanding. However, despite much progress in an amazingly short time, many questions on IFN antagonists encoded by SARS-CoV-2 still remain.

While the extend of SARS-CoV-2 proteins that inhibit IFN induction and signaling is established (Fig. 1, Table 1), the respective underlying molecular mechanism(s) often remain elusive [17–19]. Of note, most viral proteins were studied in the context of type I interferon responses. A recent study suggests that most type I IFN signaling antagonists of SARS-CoV-2 may also affect type II and III signaling, albeit to varying extend [17]. For many viral proteins, however, it remains to be determined whether they also target type II or III IFN induction and/or responses. In addition, the relevance and contribution of the individual proteins to the immune escape of SARS-CoV-2, as well as functional interactions and synergisms, are currently largely unclear. In a replicon setting, a mutant lacking Nsp1 was more sensitive toward type I IFN-mediated inhibition [26]. Furthermore, recombinant SARS-CoV-2 lacking ORF6 induces higher levels of ISGs in vitro [23]. However, more studies using recombinant viruses and in vivo models are required to better understand the individual contribution of IFN counteraction mechanisms to viral spread, replication, and pathogenesis.

SARS-CoV-2 targets IFN induction and signaling cascades at multiple levels using more than half of its proteins [17–19] (Fig. 1, Table 1). This highlights how crucial it is for successful viruses to tightly control these signaling pathways. None of the individual SARS-CoV-2 proteins inhibits the IFN system entirely. Thus, multiple factors need to synergize to allow efficient viral immune evasion and spread. Especially, IFN production and release need to be kept at a minimum by the virus to avoid setting uninfected cells in an anti-viral state and recruiting/activating the adaptive immune responses.

It was reported that SARS-CoV-1 is more resistant to inhibition by type I IFNs [51]. In vitro evidence suggested that Nsp15 of SARS-CoV-1 is a stronger type I IFN signaling antagonist than Nsp15 of SARS-CoV-2, possibly providing one explanation for the difference between the two coronaviruses [17]. How SARS-CoV-2 compares to middle east respiratory syndrome coronavirus (MERS) or seasonal coronaviruses in terms of IFN resistance is currently unknown.

SARS-CoV-2 continues to adapt for an efficient spread in the human population resulting in the emergence of variants [52, 53]. It was reported that variants of ORF3b that are either longer and shorter forms are either more or less active than the original ORF3b variant, respectively [44]. Variants of Nsp1 were detected, which show increased efficiencies in IFN antagonism [54]. It was noted, that variants of concern (VOC) differ in their resistance toward exogenous IFN, with the alpha VOC being consistently the most resistant [55, 56]. Mechanistic analysis revealed that the alpha VOC has increased relative expression levels of the IFN antagonists N, ORF9b, and ORF6 compared to an early 2020 SARS-CoV-2 strain [57].

As SARS-CoV-2 was shown to be sensitive to innate immune activation, treatment with IFNs may be beneficial in COVID-19 [58, 59]. Accordingly, the early presence of IFNs was shown to protect COVID-19 patients from severe disease [51, 60]. However, IFNs are detrimental in the long run by promoting inflammation. The excessive presence of IFNs (and other pro-inflammatory cytokines) often defines the severity of the diseases and possibly even long-term consequences of the infection [61, 62]. Consequently, anti-viral IFN therapy is usually efficient but also associated with severe side effects. Therefore, exact timing and dosing are paramount, e.g., using the most potent IFNs or synergies in combinatorial approaches with multiple different IFNs [17]. In addition, a better understanding of the mechanistic details of the IFN antagonism by SARS-CoV-2 proteins may allow us to more efficiently interfere with and perhaps even prevent viral immune evasion.

In summary, while the past more than two years have brought astounding progress in the characterization of the interplay between the IFN system and SARS-CoV-2, we are still only beginning to understand the intricate details. Many of the proteins described here also antagonize other pathways of anti-viral immunity, besides the IFN system, such as autophagy [17, 63]. Especially for therapeutic intervention, e.g., with IFNs, we need to better understand the interplay between the IFN system and SARS-CoV-2 to define the best dose, timing, and synergistic combinations of different IFNs to approach safe and effective COVID-19 therapy based on innate immune modulation [64].
